# Sexting, Online Sexual Victimization, and Psychopathology Correlates by Sex: Depression, Anxiety, and Global Psychopathology

**DOI:** 10.3390/ijerph17031018

**Published:** 2020-02-06

**Authors:** Aina M. Gassó, Katrin Mueller-Johnson, Irene Montiel

**Affiliations:** 1 Faculty of Law, Universitat Internacional de Catalunya, 08017 Barcelona, Spain; irene.montiel@unir.net; 2 Center for Criminology, Faculty of Law, Oxford University, Oxford OX1 3UL, UK; katrin.mueller-johnson@crim.ox.ac.uk; 3 Faculty of Education, Universidad Internacional de La Rioja (UNIR), 26006 Logroño, Spain

**Keywords:** sexting, mental health, psychopathology, victimization, sex

## Abstract

Recent research on sexting highlighted a relationship between this new technology-mediated behavior and psychopathology correlates, although up to date results are mixed, and so far, studies have often used simple and not clinically validated measures of mental health. This study aimed to investigate sexting behaviors, online sexual victimization, and related mental health correlates using clinically validated measures for global psychopathology, anxiety, and depression; and doing so separately for men and women. The sample consisted of 1370 Spanish college students (73.6% female; 21.4 mean age; SD = 4.85) who took part in an online survey about their engagement in sexting behaviors, online sexual victimization behaviors, and psychopathological symptomatology, measured by a sexting scale and the Listado de Síntomas Breve (brief symptom checklist) (LSB-50), respectively. Out of our total sample, 37.1% of participants had created and sent their own sexual content (active sexting), 60.3% had received sexual content (passive sexting), and 35.5% had both sent and received sexual content, with significant differences between male and female engagement in passive sexting. No differences were found between men and women in the prevalence of their victimization by nonconsensual dissemination of sexual content; however, women were more pressured and threatened into sexting than men. Sex differences in psychopathology were found only for depression prevalence rates but not for global psychopathology or anxiety. Furthermore, for male participants, our results showed a significant association only between online sexual victimization and psychopathology but not for consensual active and passive sexting. However, for the female participants, active sexting, passive sexting, and online sexual victimization were all associated with poorer mental health. Implications for prevention and intervention are discussed.

## 1. Introduction

Over the past few years, the phenomenon of sexting has received increased attention from the media and the research community as it has been linked to unwanted and harmful consequences, in particular for younger populations [1,2,3,4,5]. Sexting is generally known as the sending, receiving, or forwarding of sexual text messages, nude images, and/or sexual content (e.g., photos, videos) via the internet, mobile phones, or any electronic devices [2]. 

The scientific discourse on sexting is divided in two clear lines of argument [6]: proponents of the first line argue that sexting is a normative behavior as a part of sexual expression in relationships [7,8], whilst those of the other line of argument hold that sexting is a risky behavior that requires intervention and prevention [9,10,11]. However, Klettke et al. [12] moved towards an integrative discourse, which argues that sexting behaviors can be placed on a continuum ranging from consensual sexting behaviors as part of a normative exploratory sexual behavior, to nonconsensual or coerced sexting, associated with negative mental health consequences (online sexual victimization).

One of the main challenges of sexting research to date is that there is no consensus around its definition, and some authors use broader definitions that include any kind of erotic or sexual communication, whilst others use narrower definitions that only include image-based content [13,14,15]. Furthermore, some authors understand sexting to be only a voluntary behavior [14], whilst others include coerced sexting as part of general sexting behaviors [16]. The existing literature on sexting also differs in the population samples used for the research (teens vs adults) and in the items used to measure sexting, which contributes to the lack of a unified body of research and homogeneous results around this topic.

Accordingly, sexting prevalence rates vary considerably, ranging from 1% for participants who sent erotic or sexual content (imagery or text messages) and 7.1% for those who received the content, to 30% for participants who sent the content and 45% for those who received it in adolescent and young adult samples [16,17]. The literature review carried out by Klettke et al. [18], shows that out of all the examined studies, the mean prevalence for sexting in adults was 53.3%, and when looking only at the sending of sexts with photo content, the mean prevalence was 48.6%. More recent studies highlight similar prevalence rates in adults: Drouin et al. [19] found that 47% of their adult sample had engaged in sexting behaviors, Hudson and Fetro [20] found that 48.5% of their sample was engaging in sexting behaviors at the time they were questioned, whilst 80.9% of their sample had engaged in sexting at least once in their lifetime. Following this line of reasoning, Morelli et al. [21] found similar results in an Italian sample of ages ranging from 13 to 30 years old, in which 82.2% of the participants had engaged in sexting behaviors at least once at the moment they were questioned; Gaméz-Guadix et al.’s results showed that 66.8% of their adult Spanish sample had engaged in sexting at least once in their lifetime, and 46.7% of the sample had sexted three or more times [14]. 

Considering sex differences, research has indicated that women were more likely to engage in active sexting than men: 60.0% of females reported having sent nude photos of themselves versus 45.4% of males reported having sent nude pictures of themselves [22]. This was also the case in Gordon-Messer et al. [7], where males were more likely to receive sexts (17%) than females (8.7%). However, when these authors looked at both sending and receiving sexts, no differences between males and females were found. On the other hand, Hudson [23] reported that males engaged significantly more in sexting behaviors than females, without specifying what items were included in the sexting behaviors category. Finally, regarding online sexual victimization, Englander [16] reported that females were more likely to report being pressured to sext than males; however, they argue that this difference is explained because females have a higher reporting rate than males and not due to real differences in sexting activities. For the purposes of this study, online sexual victimization is defined as experiencing some type of pressure or threats through the Internet or mobiles phones to obtain the victim’s sexual content, or/and the dissemination by the perpetrator of sexual content without the victim’s consent [14]. 

As research has shown, there is a link between sexting and online victimization. Sexting among adults is not necessarily a criminal behavior; however, it could lead to online sexual victimization such as sextortion or nonconsensual dissemination of sexual content [4,24,25]. Among a sample of college students, those who engaged in sexting were more likely to be victimized by different types of cybervictimization [26]. Englander [27] showed that 70% of her college student sample was pressured to sext, whilst Branch et al. [28] found that approximately 10% of their sample had been victims of revenge porn (having your intimate and sexual content disseminated without consent with the intention to get revenge). In the same line, Henry et al. [29] surveyed 4274 Australian adults and reported that 1 out of 10 participants had sent sexual content to someone, and this content had then been disseminated without the person’s consent. 

In Spain, approximately 4% of 18–60 years old adults have been victims of nonconsensual dissemination of sexual content, although being pressured to sext (28.2%), being pressured to share intimate or sexual information (24.5%), being pressured or threatened to perform a sexual act on the internet (22.2%), or being threatened online to maintain sexual intercourse with someone (18.7%) were more prevalent forms of victimization than the nonconsensual dissemination of sexual content [14]. Finally, research has shown that women experience more pressure than men to create and send sexting content [27,30] and suffer more victimization from revenge porn from their partners or expartners than men [28]. 

For the purpose of this study, online sexual victimization (OSV) was investigated as part of the sexting dynamics. The online sexual victimization behaviors we measured included: being a victim of nonconsensual dissemination of nude imagery or sexual content of oneself, being pressured to sext, and being threatened to sext. Some authors have established a link between sexting and online sexual victimization and poorer mental health, indicating that sexting behaviors are associated with higher rates of depression, anxiety, substance abuse, and suicidal ideation, both in teens and in adults [4,14,16,18,26,30]. Gámez-Guadix et al. found a significant relationship between sexting and online sexual victimization, and their results indicated that women were more than two times more likely to be victimized than men [14]. Furthermore, Bendixen et al. [31] found that those students who had been subjected to nonphysical peer sexual harassment in high school showed lower psychological well-being, ranging from symptoms of depression and anxiety to self-esteem and body image. According to these authors “non-verbal peer sexual harassment may involve display of sexual pictures, and indirect harassment may involve being subject to sexual rumors and having had pictures distributed in social media” [31]. 

Regarding psychopathology, research has shown significant differences between men and women. According to Nolen-Hoeksema and Reisner et al. [32,33], women are twice as likely as men to experience depression. In surveying a Spanish population sample, Haro et al.’s results indicate that 4.37% of the population suffered from some type of depression disorder in the last year, whilst 5.71% suffered from some type of anxiety disorder in the last year [34]. Their results also showed that 6.25% of Spanish women suffered from depression disorders in the last year versus 2.33% of Spanish men, and 7.61% of Spanish women suffered from anxiety disorders versus 2.53% of Spanish men. 

Research also highlights an existing relationship between psychopathology or psychological health and online victimization behaviors, such as cyberbullying, online dating violence, revenge porn, and sexting [4,11,18,19,35,36]. In this sense, Klettke et al. [18] in their literature review found significant relationships between sexting and risky sexual behavior, with several other adverse outcomes, such as: (a) the sharing of sexual content without consent, (b) legal consequences, such as being prosecuted for child pornography distribution, and (c) negative mental health repercussions. Considering the increasing number of suicide cases related to sexting, the relationship between sexting and mental health seems of particular interest, even though current results are not homogeneous [18,30]. 

A significant association between depressive symptoms and impulsivity and sexting was found by Temple et al. [37] but the relationship was not significant when they controlled for previous sexual behaviors. Englander’s results indicate that people who engaged in sexting were less likely to have depression-related issues, but more likely to have anxiety-related problems [16]; by contrast, Van Ouytsel et al.’s findings point towards a significant relationship between depressive symptoms and engagement in sexting behaviors [38], and Gordon-Messer et al. [7] did not find significant differences in depression levels, anxiety levels, and self-esteem between subjects who had received sexts, those who had sent and received sexts, and participants who had neither sent nor received sexts. As noted in the introduction, some inconsistencies in the literature regarding the relationship between sexting and mental health variables may be related to heterogeneity in concept definition and operationalization of sexting behaviors, use of different data collection instruments and measures, use of differing survey methods, and diverse samples in terms of age range and nationalities. Moreover, these inconsistencies might appear because most empirical studies on sexting do not segregate data by sex or do not control for this variable, given that men and women relate differently to both sexting behaviors, online sexual victimization, and mental health. 

An association between sexting and emotional problems was also found in a sample of over 17,000 participants by Ševčíková [39], whilst Brinkley et al.’s results showed that sending sexts at 16 years old predicted for borderline personality traits at age 18 [40]. Finally, Gámez-Guadix and De Santisteban [41] in their longitudinal study found that depression at T1 predicted for sexting at T2, and Frankel et al. [42] found a significant relationship between consensual sexting and depressive symptoms, suicide attempts, and suicidal behaviors, even though depressive symptoms were more prevalent in participants who had reported nonconsensual sexting. 

In sum, recent research points towards an existing relationship between sexting and mental health variables; however, research has not yet examined if there might be differences in sex, regarding how sexting and psychopathology might be related.

Therefore, the general aim of this study is to analyze sexting and psychopathology correlates by sex. Considering that men and women experience sexting behaviors, online sexual victimization, and psychopathology in different ways, we hypothesize that the association between these variables will be different by sex. Therefore, the specific aims of this study are (a) to report sexting prevalence rates in a Spanish college sample by sex; (b) to analyze with what frequency college students engage in each of these sexting behaviors and online sexual victimization and if there is a difference between sex; (c) to examine psychopathology prevalence by sex using clinically validated mental health measures, and (d) to explore, separately by sex, if college students who engage in sexting behaviors and who suffer online sexual victimization have higher prevalence rates of psychopathology than those who do not engage in sexting behaviors and those who do not suffer OSV. 

## 2. Materials and Methods 

### 2.1. Participants

The sample comprised 1370 Spanish college students including 999 women (73.6%) and 359 men (26.2%) and 12 participants (0.2%) who did not specify their gender and were excluded from the analyses. The final sample comprised 1358 participants. Ages ranged from 18 to 64 years old, with a mean of 21.40 years (SD = 4.90). The descriptive statistics for the demographic variables for the total sample can be found in Table 1. 

### 2.2. Instruments

#### 2.2.1. Sexting Questionnaire

For the purpose of this research, we defined sexting as creating, sending, and/or forwarding nude or sexually explicit images or videos through any electronic device (i.e., excluding text messages), and online sexual victimization is defined as experiencing some type of pressure or threats through the Internet or mobiles phones to obtain the victim’s sexual content, or/and the dissemination by the perpetrator of sexual content without the victim’s consent [14].

We used a modified version of the Juvenile Online Victimization Questionnaire (JOV-Q) to assess five different types of sexting behaviors [43]. For each of the measured sexting behaviors, we asked how many times they engaged in the behavior in the past year. This then was recoded as lifetime prevalence (yes, at least once/no, never engaged in this behavior). Sexting behaviors were categorized into active sexting and passive sexting. Active sexting referred to creating and sending nude pictures of oneself or sexual content; passive sexting included receiving unsolicited sexts, and online sexual victimization (OSV) included (a) being a victim of nonconsensual dissemination of nude images of oneself or sexual content, (b) being pressured to sext, and (c) being threatened to sext. New measures were also created to asses global sexting behaviors: active–passive overlap sexting encompasses only those people who engaged in both active and passive sexting, and any sexting behavior includes all of the participants who engaged at least once in any of the measured sexting behaviors. 

#### 2.2.2. Mental Health Questionnaire

In order to measure mental health, we used the Spanish version of the Listado de Síntomas Breve (brief symptom checklist), which is a revised and shorter version of the SCL-90 [44]. This instrument consists of 50 items that assess psychopathological symptomatology. Responses to the items were collected on a 4-point Likert scale (0 = never and 4 = extremely). We used the global subscale, the depression subscale, and the anxiety subscale for this study. To analyze the presence or absence of mental health symptoms, the results obtained from the LSB-50 questionnaire were converted according to the authors guidelines [44]. All scores under 85 were considered as symptomatology not present and were given a 0, and those who obtained 85 or higher were considered as symptomatology present and were given a 1. This threshold was used following author’s guidelines [44]. For the purpose of this study, psychopathology will be defined as the manifestation of behaviors and experiences, which may be indicative of mental illness or psychological impairment. 

#### 2.2.3. Socio-Demographic Questionnaire

We included questions about age, sex, marital status, parental marital status, place of residence, employment situation, academic situation, and questions about frequency and use of phones and social media. 

### 2.3. Procedure

The study was approved by the Ethics Committee of the International University of Catalunya (UIC Barcelona). The ethical approval code is DRET-2018-02. Participation was voluntary, and responses were anonymous to promote openness and honesty. The survey was administered online. The survey link was sent to university professors from Spanish universities with a request to pass it on to their students. The participating students then self-selected to take part in their own time. The questionnaire took approximately 20–25 min to complete, and once completed, students were given information on community resources in case of distress and the email address to contact the investigators in case of concerns. No participant contacted the investigators. This paper is a first foray into the collected data. Through the survey administered to participants, we collected more data than disclosed in this paper. However, the additional data were not included in this paper because we understand they address different issues than the ones we have tried to explore in the present study. Further data will be available and published in forthcoming papers.

## 3. Results

### 3.1. Sample Demographic Characteristics

Table 1 shows the descriptive statistics of demographic and background variables for the total sample, men, and women. In the sample, 54.6% of the participants were single, 92.4% were undergraduate students, and the greater majority were living with their parents (62.4%) and did not hold any job in addition to being a student (67.4%). Out of the total sample, 98% of participants owned a smartphone and 97.8% used social media, using their mobile phones as the most frequent form of internet access (89.8%). The mean age of having their first phone was 13.9 years old, and the mean age for the first internet use was 12.01 years old. Finally, the greater majority of participants used the internet more than 3 h per day (48.0%).

### 3.2. Prevalence and Frequency of Sexting Behaviors and Online Sexual Victimization (OSV) by Sex

The prevalence of the measured sexting behaviors for the total sample and by sex are shown in Table 2. For the active sexting behaviors, 37.1% of participants had created and sent nude images of themselves or sexual content to someone voluntarily. There was no sex difference in this. 

For passive sexting behavior, prevalence rates for receiving sexts was 60.3% for the total sample; however, men were 1.45 times more likely to receive sexts than women. 

The prevalence rates for OSV showed that for being a victim of nonconsensual dissemination of sexting, 3.3% of the total sample reported having been victimized, and no differences were found between sex. However, our results showed that 37.1% of women reported being pressured to sext, in comparison to 19.2% of men. For this victimization behavior, women were 2.49 times more likely to be pressured to sext than men, and they were 5.06 times more likely to be threatened to sext than men (4.4% vs 0.9%). 

Finally, for the global measures of sexting, for the active–passive sexting overlap, our results showed that 35% of the participants engaged in both active and passive sexting, with men more likely than women to engage in both behaviors (*p* = 0.000, OR = 1.80). Ultimately, 72% of the sample reported ever engaging in any of the sexting behaviors, with a closely equal participation between men and women.

We next analyzed the frequency of the individual sexting behaviors for both men and women in order to see if there were any differences between the two groups (Table 3). The most common sexting behavior appeared to be receiving sexts 2–3 times in the last year for both men (37%) and women (33.6%). For the active sexting behavior, our results showed that there were no differences between sex for creating and sending sexual content. 

For the passive sexting behavior, significant differences were found between men and women, with males receiving sexts more frequently (z = −4.373, *p* = 0.000) than females. No significant differences were found between males and females for the online sexual victimization item of being a victim of nonconsensual dissemination of sexting, but most of the victims reported being victims 1 time (2.8% women; 1.2% men) or 2–3 times (0.5% women, 2.1% men) in the last year. For being pressured to sext and being threatened to sext, significant differences were found between sex. Women were more likely to be more frequently pressured (z = 6.054, *p* = 0.000) and threatened to sext (z = 3.000, *p* = 0.003) than men, with the most frequent form of victimization being to be pressured to sext 2–3 times in the last year for female participants (23.2% vs 10.9%). 

Finally, for the global measures of sexting, significant differences were found between male and female for the frequency of those who engage in both active and passive sexting, with men (45.4%) reporting higher sexting frequency rates than women (31.6%). No significant differences were found for the frequency of engaging in any sexting behavior between sex. 

### 3.3. Prevalence of Psychopathology by Sex

The prevalence rates of psychopathology for the total sample and by sex are shown in Table 4. Our results indicate that, using the standard threshold for the LSB-50, almost 40% of participants out of the total sample presented global psychopathology, almost 50% of participants suffered from anxiety, and almost 30% suffered from depression. Looking at the differences between sex, our results show that there were no significant differences between males and females for presenting global psychopathology, nor for anxiety. However, results showed a significant difference between men and women for suffering from depression, with men being 1.46 times more likely to present it than women. 

### 3.4. Association between Psychopathology and Sexting Behaviors by Sex

Furthermore, we investigated the relationship between psychopathology and the different types of sexting behaviors and online sexual victimization for men and women separately. Results are presented in Table 5. Our results showed that, for men, psychopathology prevalence rates were higher for those participants who engaged in sexting behaviors than for those who did not engage in sexting behaviors, however not significantly so. No significant differences in any of the psychopathology measures were found for the active sexting behavior nor for the passive sexting behavior. Regarding online sexual victimization, men who reported being victims of nonconsensual dissemination of sexting were 5.54 times more likely to present global psychopathology than those who did not report being a victim of nonconsensual dissemination of sexting. No significant differences were found between the rest of online sexual victimization and psychopathology for the male sample. Finally, male participants who reported engaging in both active and passive sexting behaviors reported significantly higher prevalence rates for depression than those participants who did not engage in both active and passive sexting behaviors.

On the other hand, for the female sample, results established a relationship between active sexting and psychopathology. More specifically, females who had created and sent their sexual content were 1.47 times more likely to show global psychopathology and were 1.63 times more likely to suffer from depression than the female participants who had not engaged in active sexting. Significant differences were found for all of the psychopathology measures and the passive sexting behavior. More specifically, women who received sexts were 1.53 times more likely to present global psychopathology, 1.53 times more likely to report anxiety, and 1.43 times more likely to show depression than women who had not received sexts. Furthermore, female participants who had been victims of nonconsensual dissemination of their sexual content were 2.60 times more likely to show global psychopathology, 2.20 times more likely to show anxiety, and 2.95 times more likely to show depression than those women who had not been victims of nonconsensual dissemination of sexting. Moreover, those female students who had been pressured to sext reported higher prevalence rates for all of the psychopathology measures, being 1.56 times more likely to present global psychopathology, 1.55 times more likely to report anxiety, and being 2.08 times more likely to suffer from depression. Those women who reported being threatened to sext were 3.04 times more likely to suffer from global psychopathology, 2.12 times more likely to suffer from anxiety, and 1.85 times more likely to suffer from depression than women who had not been threatened to sext. 

Finally, for the global sexting behaviors, results showed that women who engaged in both active and passive sexting were 1.64 times more likely to show global psychopathology, 1.51 times more likely to present anxiety, and 1.91 times more likely to report depression that those who did not engage in both behaviors. Ultimately, for those women who reported engaging in any type of sexting behavior, prevalence rates were higher for the three psychopathology measures (global, anxiety, and depression) than for those women who did not report engaging in any type of sexting behavior and online sexual victimization. 

## 4. Discussion

Due to the rapid development of new technologies, new ways of social and romantic interactions have appeared. One of these new ways of social interaction is sexting. Current research shows that consensual and voluntary sexting among adults is becoming part of a normal sexual expression [6,12]; however, sexting has been associated with different types of victimization and is understood by many authors to be a risky behavior as it increases the chances of suffering sexual victimization [6,8,9]. Considering that men and women experience sexting behaviors and mental health in different ways, we hypothesized that the association between sexting and mental health would be different for men and women, so the general aim of this study was to analyze this issue. Therefore, the specific aims of this study were to report sexting prevalence rates by sex, to analyze with what frequency college students engaged in each of the sexting behaviors and suffered from online sexual victimizations and if there was a difference between sex, and to examine psychopathology prevalence by sex. Finally, this study aimed at exploring if college students who engage in sexting behaviors and who suffer online sexual victimization have higher prevalence rates of psychopathology than those who do not engage in sexting behaviors and those who do not suffer OSV, by sex.

Overall, our results showed that more than one third of college students had engaged at least once in the past year in active sexting, consistently with the results obtained by many studies with adult and college samples, in which prevalence rates range from 27.8% to 49% for this behavior [7,14,16,21,42,45,46,47]. For the passive sexting behavior, our results showed that out of our total sample, almost two thirds of participants had received sexts at least once in the past year, in line with evidence found in other studies, with their prevalence rates between 54.3% and 64.2% for this behavior [18,48,49].

No significant differences between prevalence rates for male and female participants were found for the active sexting behavior (creating and sending sexual content), in line with results found by Benotsch et al., Dir et al., Drouin and Landgraff, Gordon-Messer et al., Hudson et al., Gámez-Guadix et al., and Klettke et al. [3,7,12,14,23,49,50]. However, our results are in direct contradiction to those found by AP-MTV and Englander, whose findings indicated that females were more likely to send sexts than male [16,45]. As Englander suggests, the differences in prevalence rates found between men and women for this study might be due to different reporting rates, with girls more likely to report being pressured, coerced, blackmailed, or threatened into sexting than males [16]. 

Significant differences were found between men and women for prevalence rates of passive sexting behavior (receiving sexts), indicating that males are more likely to receive sexts than females, corroborating the results showed by AP-MTV [7,12,45,49]. As Gordon-Messer et al. [7] point out, these differences found between males and females might be attributable to the fact that males are more used to receiving sexual content from their peers without sending content back and more used to pressuring women to sext and thus, to receiving their sexts. 

With regard to the OSV, our results showed that 3.3% of the total sample had been a victim of nonconsensual dissemination of their sexual content, in line with Gámez-Guadix et al.’s findings [14] and further away from Henry et al.’s results [51], which state that around 11% of their Australian sample (16–49 years old) were victims of nonconsensual dissemination of their sexual content. These differences in prevalence rates might be explained by cultural differences or a broad age range in Henry et al.’s sample [51].

Moreover, one out of three participants from our sample had been victimized by being pressured to sext, and 3.4% had been victimized by being threatened into sexting. Gámez-Guadix et al. [14] found similar results, with 28.2% of the total sample being pressured to send sexual pictures, 3.3% being victimized by nonconsensual dissemination of sexting images, and 1.9% of the total sample being threatened into send sexual pictures. However, our results could vary when considering the participant’s sexual orientation, as Bendixen et al.’s work suggests [52], but we did not control for sexual orientation in the present study. 

With regard to sex, no significant difference was found between men and women for the OSV item of being a victim of nonconsensual dissemination of their sexual content, but, by contrast, women were more likely to be pressured to sext than men, in line with Gámez-Guadix et al. [14] and with Henry et al. [51]. Festl et al. [53] surveyed 1033 German internet users (14–20 years old) with regard to online sexual victimization and found that women suffered from more victimization experiences than men. These results indicate that, even though both men and women experience online sexual victimization, rates are higher for women, as offline sexual victimization literature has also shown [54,55].

Similarly to our results, Dir et al.’s findings showed that most college students engage in sexting behaviors only occasionally or rarely and that those who engage in sexting behaviors weekly or daily are a rare minority [49]. Our results indicated that women experience higher prevalence rates of being pressured and being threatened to sext, with a higher frequency than men. 

Our results confirmed a difference in psychopathology prevalence rates between male and female for depression, although in the opposite direction to what we expected. Our results regarding psychopathology prevalence showed that men were more likely to suffer from depression than women and showed no significant differences between male and female for anxiety and global psychopathology. We expected to find women to be more likely to suffer from depression than men, and these results disconfirmed our expectations. One reason for these results might be explained by self-selection among the men who took part in the survey, meaning that men who are depressed might be more likely to take part in the survey than men who are not depressed. These results are in line with Klettke et al. [12], who found depressive symptoms to be more prevalent amongst men than women. However, our findings are contrary to other literature findings, where significant differences between male and female have been found. According to Nolen-Hoeksema and Reiser et al., women are twice as likely as men to experience depression [32,33]. Haro et al.’s results indicated that anxiety disorders were more prevalent than depression disorders [34]. Furthermore, results showed that Spanish women were almost three times as likely to suffer from depression disorders in the past year than men, and they were three times more likely to report anxiety disorders than men [34]. Bendixen et al. found that nonphysical peer sexual harassment had an evident adverse effect on the subject’s well-being for both genders but affected women’s depressive symptoms more in both of the conducted studies [31]. 

Regarding the association between sexting and psychopathology in men, we did not find significant differences between those who engaged in active sexting behaviors and those who did not, for any of the psychopathology measures. However, males who had been victims of nonconsensual dissemination of their sexual content showed higher rates of global psychopathology than those who were not victims. These higher psychopathology rates for this behavior suggest that for males, psychopathology is not related to consensual sexting behaviors nor to being pressured or threatened to sext, but only to suffering victimization by nonconsensual dissemination of sexual content. 

On the other hand, we did find an association between sexting behaviors and psychopathology for the female sample. Our results showed that for women, creating and sending their own sexual content was related to higher global psychopathology and depression prevalence rates than for women who did not engage in this behavior. People who suffer from depressive symptoms might lack coping strategies when they are pressured by their peers to create and send sexual content, resulting in a higher engagement in coercive sexting [15,31]. 

However, our results regarding the female sample are contrary to Klettke et al.’s results [12], showing that not only nonconsensual and unwanted sexting are associated with poorer mental health, but in women it is also related to consensual active sexting. One of the reasons for this discrepancy might be due to the fact that when we asked participants if they had created and sent their own sexual content we did not specify it had to be voluntarily, so some of the female participants who have responded affirmatively to being pressured to sext might be the same ones who have responded affirmatively to creating and sending their own sexual content. This would explain why in our study female participants showed a relationship between active sexting and poorer mental health. 

Furthermore, for female participants, receiving sexts was associated with higher prevalence rates for all of the psychopathology measures than those women who had not received sexts. These findings suggest that for women receiving unwanted and unsolicited sexual content might be a distress-generating factor, as it is possible that they perceive this action as a form of indirect sexual harassment [31]. Similarly, women who had been victims of online sexual victimizations also reported higher psychopathology prevalence rates for all of the psychopathology measures than those women who had not been victims of online sexual victimization. These results show that although active sexting is associated with higher rates of global psychopathology and depression, receiving sexts and victimizing sexting behaviors are associated with more psychopathological symptoms than active sexting. These results could be explained by victimizing behaviors triggering greater psychopathological symptomatology or by women who suffer from psychopathology, anxiety, or depression being more vulnerable to being pressured to sext and to different forms of online sexual victimization [41]. The negative consequences of these behaviors are intimately related with gender, since women experience more negative outcomes due to gender myths and traditional expectancies regarding sexual norms for women in particular [56]. A qualitative study regarding emotional and mental health outcomes of nonconsensual dissemination of intimate images in revenge porn carried out by Bates reveals a higher presence of post-traumatic stress disorder, anxiety, depression, and suicidal ideation in women, finding similar consequences to physical sexual aggressions [57]. 

Our results indicate that the relationship between sexting and psychopathology is different for men and women. In this sense, for men, poorer mental health is associated only to victimization by nonconsensual dissemination of sexual content, whilst for women, poorer mental health is associated with all of the sexting behaviors and online sexual victimizations. The inconsistencies in the literature regarding the relationship between sexting and mental health could be partially explained by those studies not having considered that males and females engage and respond differently to sexting behaviors and online sexual victimization. Our evidence shows that there is a strong relationship between sexting and psychopathology; however, this relationship is not equal for men and women, with females being more vulnerable to sexting, online sexting victimizations, and psychopathology. These results contribute to a deeper understanding of how men and women relate to sexting behaviors and online sexual victimizations, in order to design effective sexting and mental health prevention and intervention campaigns. In this line, our results point out that mental health practitioners might find it interesting to look into online sexual victimization experiences in males who suffer from psychopathology and look into sexting behaviors and online victimization experiences in females who suffer from psychopathology, anxiety, and depression as a possible cause for their psychological distress. 

This study has several limitations that should be considered when interpreting the results. First, the sample used was nonprobabilistic and comprised of only college students, rather than the general population, so generalization of results should be cautiously done. In this sense, the sample used was self-selected using an online survey, which would explain why the total sample is unbalanced regarding female and male participants. Taking this into account, data analysis was conducted separately for males and females, in order to decrease the impact of the sample bias. Furthermore, the female sample was three times bigger than the male sample, which might explain why results regarding depression scores were higher for males than for females. It is possible that males who decided to participate in the survey were randomly males with higher depression rates, and thus the interpretation of the results should be extracted cautiously. Furthermore, sexual orientation was not accounted for when carrying out the data analysis, and it should be stated that results regarding online sexual victimization could have varied if considering this variable, as regarded by Bendixen et al. [52]. Second, this study is cross-sectional, and not longitudinal, so no temporal relationships can be established between mental health variables and sexting behaviors. Finally, in order to increase cross measurement validity of findings, other studies should try to replicate our results obtained with a particular, clinically validated, psychometric questionnaire, with other mental health instruments.

## 5. Conclusions

In conclusion, this is the first study to examine the relationship between sexting behaviors, online sexual victimization, and psychopathology by sex using clinically validated mental health measures amongst a Spanish college sample. We hypothesized that sexting behaviors and online sexual victimization prevalence rates, and similarly, psychopathology rates, would be different for males and females, and thus, we expected the relationship between sexting and psychopathology to be different for men and women. 

As the body of research regarding sexting keeps growing, more findings highlight that it is not necessarily a deviant behavior [58,59]; however, they point towards an association between nonconsensual or coerced sexting and risky behaviors, negative consequences, and poorer mental health [4,12,59,60]. Our results contribute to a deeper understanding of the relationship between sexting behaviors, online sexual victimization and psychopathology, anxiety, and depression, especially considering sex differences. Our evidence suggests that online sexual victimization is associated with poorer mental health for both men and women; however, for females, poorer mental health is also associated with consensual sexting (sending sexts and receiving sexts). These findings can be useful when designing prevention and intervention strategies, for the educational community and mental health practitioners. When interacting with young men with psychopathology symptoms and women with psychopathology symptoms, anxiety, and depression, mental health professionals might find it interesting to inquire about online sexual victimization experiences and the engagement in sexting behaviors. Further research should also explore if there are differences in mental health between consensual and nonconsensual sexters and should analyze the relationship between sexting and nonconsensual dissemination of sexual content.

## Figures and Tables

**Table 1:** Descriptive statistics of demographic and background variables.

Demographic Variables	Total Sample % (*N* = 1370)	Men % (*N* = 359)	Women % (*N* = 999)
**Sex**			
Male	26.40		
Female	73.60		
**Age**	M = 21.43 (SD = 4.85)	M = 21.98 (SD = 5.51)	M = 21.23 (SD = 4.58)
**Marital Status**			
Single	54.60	61.80	52.10
In Relationship	42.00	33.70	44.80
Married	1.20	1.40	1.20
Common Law Partner	1.30	1.70	1.20
Divorced/Separated	0.90	1.40	0.70
**Parental Marital Status**			
Married	71.30	74.70	70.40
Divorced/Separated	22.50	17.60	23.90
Widow	4.40	5.10	4.10
Other	1.80	2.60	1.50
**Academic Situation**			
Undergraduate	92.40	94.10	91.70
Master’s Degree	4.00	2.50	4.50
Erasmus	1.50	.80	1.70
Other	2.20	2.50	2.10
**Living Situation**			
With Parents	62.40	71.10	59.10
Student Apartment	22.40	15.60	24.90
Off Campus Student Residence	4.60	3.40	4.90
On Campus Student Residence	0.70	0.60	0.80
Alone	3.80	4.20	3.50
With Partner	6.20	5.10	6.70
**Employment Status**			
Unemployed	67.40	65.70	67.90
Employed Full Time	5.10	7.30	4.30
Employed Partial Time	27.40	27.00	27.70
**Own Smartphone**	98.00	98.60	
**Age of First Phone**	M = 13.86 (SD = 3.42)	97.80	
**Age of First Internet Access**	M = 12.01 (SD = 3.83)		
**Internet Access**			
Mobile Phone	89.80	81.60	92.70
Laptop	27.80	26.50	28.30
Desktop PC	6.00	13.40	3.40
Tablet	30.90	27.70	32.50
PlayStation	5.70	7.00	5.30
**Frequency Internet Access**			
Once a Week	0.10	0.30	0
2–3 Times a Week	0.40	0.60	0.30
Everyday	33.00	33.00	32.90
2–3 h per Day	16.70	16.50	16.60
More than 3 h per Day	48.00	47.60	48.40
**Social Media Use**	97.80	96.60	98.20

M: Mean; SD: Standard Deviation.

**Table 2:** Prevalence of sexting behaviors by sex.

Behaviors	Total Sample % (*N* = 1370)	Men % (*N* = 359)	Women % (*N* = 999)	Sig. Test, OR
Active Sexting Behavior				
Creating and sending nude or sexual imagery of oneself	37.1	36.5	36.9	χ^2^ (1, *n* = 1325) = 0.19, *p* = 0.890, OR = 1.018, 95% CI [0.79, 1.31]
Passive Sexting Behavior				
Receiving sexts	60.3	66.9	58.2	χ^2^ (1, *n* = 1313) = 7.96, *p* = 0.005, OR = 1.45, 95% CI [1.12, 1.88]
Online Sexual Victimization				
Being a victim of nonconsensual dissemination	3.3	3.2	3.3	χ^2^ (1, *n* = 1298) = 0.007, *p* = 0.935, OR = 0.97, 95% CI [0.48, 1.95]
Being pressured to sext	32.7	19.2	37.1	χ^2^ (1, *n* = 1312) = 36.9, *p* = 0.000, OR = 2.49, 95% CI [1.84, 3.36]
Being threatened to sext	3.4	0.9	4.4	χ^2^ (1, *n* = 1299) = 8.96, *p* = 0.003, OR = 5.06, 95% CI [1.56, 16.44]
Global Sexting Behaviors				
Active–passive sexting overlap	35.5	45.5	31.6	χ^2^ (1, *n* = 1358) = 21.9, *p* = 0.000, OR = 1.80, 95% CI [1.40, 2.30]
Any sexting behavior	72.0	73.8	71.4	χ^2^ (1, *n* = 1358) = 0.78, *p* = 0.376, OR = 1.13, 95% CI [0.86, 1.48]

OR: Odds Ratio; CI: Confidence Interval.

**Table 3:** Distribution of frequencies of sexting behaviors in percentages by sex.

Behaviors	Women % (*N* = 999)						Men % (*N* = 359)						Sig. Test (Mann–Whitney U-Test)
	0	x1	x2–3	x1–2 Month	x1–2 Week	x1 Day	0	x1	x2–3	x1–2 Month	x1–2 Week	x1 Day	
Active Sexting Behavior													
Creating and sending nude or sexual imagery of oneself	63.1	7.3	18.9	7.3	3.3	0.1	63.5	7.0	21.4	4.9	2.9	0.3	z = 0.320, *p* = 0.749
Passive Sexting Behavior													
Receiving sexts	41.8	16.0	33.6	6.4	1.9	0.1	33.1	12.7	37.0	9.5	5.3	2.4	z = −4.373, *p* = 0.000
Online Sexual Victimization													
Being a victim of nonconsensual dissemination	96.7	2.8	0.5	0.00	0.00	0.00	96.8	1.2	2.1	0.00	0.00	0.00	z = 0.035, *p* = 0.972
Being pressured to sext	62.9	10.7	23.2	2.1	0.7	0.4	80.8	6.5	10.9	1.2	0.6	0.00	z = 6.054, *p* = 0.000
Being threatened to sext	95.6	2.0	2.2	0.1	0.00	0.1	99.1	0.6	0.3	0.00	0.00	0.00	z = 3.000, *p* = 0.003
Global Sexting Behaviors													
Active–passive sexting overlap	68.4	31.6	0.00	0.00	0.00	0.00	54.6	45.4	0.00	0.00	0.00	0.00	z = −4.682, *p* = 0.000
Any sexting behavior	28.6	71.4	0.00	0.00	0.00	0.00	26.2	73.8	0.00	0.00	0.00	0.00	z = −0.885, *p* = 0.376

**Table 4:** Prevalence of psychopathology by sex.

Psychopathology	Total Sample % (*N* = 1370)	Men	Women	Sig. Test, OR
IGS	39.9	43.1	38.8	χ^2^ (1, *n* = 1322) = 1.97, *p* = 0.160, OR = 1.19, 95% CI [0.93, 1.53]
Anxiety	49.6	49.7	49.7	χ^2^ (1, *n* = 1322) = 0.000, *p* = 0.995, OR = 1.00, 95% CI [0.78, 1.28]
Depression	29.9	35.9	27.7	χ^2^ (1, *n* = 1322) = 8.23, *p* = 0.004, OR = 1.46, 95% CI [1.23, 1.90]

IGS: Global Symptomatology Index.

**Table 5:** Prevalence of psychopathology by sexting behaviors and online sexual victimization.

Men				
Behaviors		IGS (%)	Anxiety (%)	Depression (%)
**Active sexting behavior**				
Creating and sending nude or sexual imagery of oneself	No	41.3	47.9	33.8
	Yes	46.0	53.2	38.7
	Sig. Test, OR	χ^2^ (1, *n* = 337)= 0.692, *p* = 0.405, OR = 1.21, 95% CI [0.77, 1.89]	χ^2^ (1, *n* = 337) = 0.893, *p* = 0.345, OR = 1.24, 95% CI [0.79, 1.93]	χ^2^ (1, *n* = 337) = 0.823, *p* = 0.364, OR = 1.24, 95% CI [0.78, 1.96]
**Passive sexting behavior**				
Receiving sexts	No	40.0	50.9	30.9
	Yes	44.8	50.2	38.9
	Sig. Test, OR	χ^2^ (1, *n* = 331) = 0.689, *p* = 0.407, OR = 1.22, 95% CI [0.76, 1.94]	χ^2^ (1, *n* = 331) = 0.014, *p* = 0.907, OR = 0.97, 95% CI [0.62, 1.54]	χ^2^ (1, *n* = 331) = 2.04, *p* = 0.154, OR = 1.42, 95% CI [0.88, 2.32]
**Online sexual Victimization**				
Being a victim of nonconsensual dissemination	No	41.9	49.7	34.8
	Yes	80.0	70.0	60.0
	Sig. Test, OR	χ^2^ (1, *n* = 332) = 5.73, *p* = 0.017, OR = 5.54, 95% CI [1.16, 26.51]	χ^2^ (1, *n* = 332) = 1.60, *p* = 0.206, OR = 2.36, 95% CI [0.60, 9.30]	χ^2^ (1, *n* = 332) = 2.69, *p* = 0.101, OR = 2.81, 95% CI [0.78, 10.17]
Being pressured to sext	No	42.7	49.1	35.6
	Yes	46.2	56.9	36.9
	Sig. Test, OR	χ^2^ (1, *n* = 332) = 0.254, *p* = 0.641, OR = 1.15, 95% CI [0.68, 1.98]	χ^2^ (1, *n* = 332) = 1.29, *p* = 0.256, OR = 1.37, 95% CI [0.79, 2.37]	χ^2^ (1, *n* = 332) = 0.041, *p* = 0.840, OR = 1.06, 95% CI [0.60, 1.86]
Being threatened to sext	No	43.3	50.6	35.6
	Yes	66.7	66.7	66.7
	Sig. Test, OR	χ^2^ (1, *n* = 329) = 0.663, *p* = 0.415, OR = 2.62, 95% CI [0.24, 29.23]	χ^2^ (1, *n* = 329) = 0.306, *p* = 0.580, OR = 1.95, 95% CI [0.18, 21.73]	χ^2^ (1, *n* = 329) = 1.25, *p* = 0.264, OR = 3.62, 95% CI [0.32, 40.36]
**Global sexting behaviors**				
Active–passive sexting overlap	No	40.2	49.2	36.7
	Yes	46.5	50.3	42.1
	Sig. Test, OR	χ^2^ (1, *n* = 348) = 1.41, *p* = 0.235, OR = 1.29, 95% CI [0.85, 1.98]	χ^2^ (1, *n* = 348) = 0.042, *p* = 0.837, OR = 1.05, 95% CI [0.69, 1.59]	χ^2^ (1, *n* = 348) = 4.92, *p* = 0.027, OR = 1.64, 95% CI [1.06, 2.56]
Any sexting	No	37.1	41.6	29.2
	Yes	45.2	52.5	38.2
	Sig. Test, OR	χ^2^ (1, *n* = 348) = 1.77, *p* = 0.183, OR = 1.40, 95% CI [0.85, 2.29]	χ^2^ (1, *n* = 348) = 3.17, *p* = 0.075, OR = 1.55, 95% CI [0.95, 2.53]	χ^2^ (1, *n* = 348) = 2.34, *p* = 0.126, OR = 1.50, 95% CI [0.89, 2.52]
**Women**				
	%	IGS	Anxiety	Depression
**Active sexting behavior**				
Creating and sending nude or sexual imagery of oneself	No	35.2	48.3	23.7
	Yes	44.5	51.3	33.7
	Sig. Test, OR	χ^2^ (1, *n* = 955) = 8.05, *p* = 0.005, OR = 1.47, 95% CI [1.13, 1.93]	χ^2^ (1, *n* = 955) = 0.767, *p* = 0.381, OR = 1.12 95% CI [0.86, 1.46]	χ^2^ (1, *n* = 955) = 11.08, *p* = 0.001, OR = 1.63, 95% CI [1.22, 2.18]
**Passive sexting behavior**				
Receiving sexts	No	33.1	43.6	23.6
	Yes	43.1	54.2	30.6
	Sig. Test, OR	χ^2^ (1, *n* = 954) = 9.73, *p* = 0.002, OR = 1.53, 95% CI [1.17, 2.00]	χ^2^ (1, *n* = 954) = 10.48, *p* = 0.001, OR = 1.53, 95% CI [1.18, 1.99]	χ^2^ (1, *n* = 954) = 5.80, *p* = 0.016, OR = 1.43, 95% CI [1.07, 1.92]
**Online sexual victimization**				
Being a victim of nonconsensual dissemination	No	37.8	48.8	26.6
	Yes	61.3	67.7	51.6
	Sig. Test, OR	χ^2^ (1, *n* = 938) = 6.97, *p* = 0.008, OR = 2.60, 95% CI [1.25, 5.43]	χ^2^ (1, *n* = 938) = 4.28, *p* = 0.038, OR = 2.20, 95% CI [1.02, 4.72]	χ^2^ (1, *n* = 938) = 9.45, *p* = 0.002, OR = 2.95, 95% CI [1.44, 6.05]
Being pressured to sext	No	34.7	45.5	22.0
	Yes	45.4	56.3	36.9
	Sig. Test, OR	χ^2^ (1, *n* = 951) = 10.58, *p* = 0.001, OR = 1.56, 95% CI [1.19, 2.04]	χ^2^ (1, *n* = 951) = 10.51, *p* = 0.001, OR = 1.55, 95% CI [1.19, 2.02]	χ^2^ (1, *n* = 951) = 24.82, *p* = 0.000, OR = 2.08, 95% CI [1.55, 2.77]
Being threatened to sext	No	37.2	48.5	26.9
	Yes	64.3	66.7	40.5
	Sig. Test, OR	χ^2^ (1, *n* = 943) = 12.47, *p* = 0.000, OR = 3.04, 95% CI [1.59, 5.80]	χ^2^ (1, *n* = 943) = 5.30, *p* = 0.021, OR = 2.12, 95% CI [1.10, 4.09]	χ^2^ (1, *n* = 943) = 3.74, *p* = 0.053, OR = 1.85, 95% CI [0.98, 3.49]
**Global sexting behaviors**				
Active–passive sexting overlap	No	35.0	48.6	23.5
	Yes	46.9	52.1	36.9
	Sig. Test, OR	χ^2^ (1, *n* = 974) = 12.56, *p* = 0.000, OR = 1.64, 95% CI [1.25, 2.16]	χ^2^ (1 ,*n* = 974) = 1.05, *p* = 0.085, OR = 1.51, 95% CI [0.88, 1.51]	χ^2^ (1, *n* = 974) = 19.00, *p* = 0.000, OR = 1.91, 95% CI [1.42, 2.56]
Any sexting	No	29.2	43.1	22.6
	Yes	42.6	52.3	29.7
	Sig. Test, OR	χ^2^ (1, *n* = 974) = 14.83, *p* = 0.000, OR = 1.80, 95% CI [1.33, 2.43]	χ^2^ (1, *n* = 974) = 6.70, *p* = 0.010, OR = 1.45, 95% CI [1.09, 1.92]	χ^2^ (1, *n* = 974) = 4.94, *p* = 0.026, OR = 1.45, 95% CI [1.04, 2.00]
